# Physical Activity Among Adults With Arthritis [Letter]

**DOI:** 10.5888/pcd11.140025

**Published:** 2014-02-20

**Authors:** Kristina A. Theis, Charles G. Helmick, Jennifer M. Hootman, Kamil E. Barbour

## To the Editor:

“Health Care Providers’ Recommendations for Physical Activity and Adherence to Physical Activity Guidelines Among Adults With Arthritis” by Austin et al (1) addresses an important area and reports promising findings related to physical activity among adults with arthritis. The Arthritis Program of the Centers for Disease Control and Prevention (CDC) is interested in this area of research, and we feel that numerous critical issues need to be addressed.

First, it should be clarified that, although the American College of Rheumatology recommends physical activity for adults with osteoarthritis (2) and the *2008 Physical Activity Guidelines for Americans* (www.health.gov/paguidelines) has guidelines for all adults (including those with arthritis), neither provide a specific guideline for adults with arthritis as indicated in the article.

Second, the authors state that “the 2011 BRFSS [Behavioral Risk Factor Surveillance System] arthritis management module was administered in 5 US states”; however, 11 states used the module (data release: www.cdc.gov/brfss/annual_data/annual_2011.htm). Although ascertaining which states used specific modules in the BRFSS is admittedly complex, this oversight is regrettable in that data on more than half the relevant states were unused.

Third, there are objective errors or missing information. For example, it is unclear how the authors obtained estimates for meeting physical activity recommendations because the 2011 BRFSS physical activity questions (3) did not ask about “physical activity in a usual week,” and CDC did not provide a variable called “adherence to recommended levels of physical activity” based on arthritis-specific recommendations. The recall period for the 2011 physical activity questions was “past month” or times “per week” or times “per month,” so direct calculation of the number of minutes exercised per day per week is not possible. Therefore, the dependent variable (adherence to physical activity guidelines) is not characterized in terms of what it actually represents. Detailed guidance on analyzing the BRFSS physical activity variables is available (www.cdc.gov/brfss/pdf/PA%20RotatingCore_BRFSSGuide_508Comp_07252013FINAL.pdf).

Other unclear analysis-related issues include identifying the specific data files used to create the analytic data set, definitions of control variables, and results of reported testing for multicollinearity. Also, the authors claim pain is an important attribute but do not explain why they chose not to use the available data on the pain variable.

Fourth, unweighted (10,908) and weighted (10,892) sample sizes should not be nearly identical if correct weights and analyses were used. The weighted sample size should reflect the population that the sample represents; if sampling weights were not used, the results are not valid. It is also unclear if the complex sampling design was taken into account.

Fifth, problematic variable parsing leads to counterintuitive results. For example, to be “unemployed” a person must not have a job *and* be looking for a job (www.bls.gov/cps/lfcharacteristics.htm#unemp); not being employed is not equivalent to being unemployed. However, the authors have lumped everyone who is not employed together, introducing confounding about this heterogeneous group, which includes students and homemakers who may be younger, more fit, or more likely to exercise than those who are unemployed.

Finally, the study is not contextualized in terms of the literature: outdated references (eg, references 4 and 5 in the Austin et al article) are used, and several relevant quantitative (4,5) and qualitative (6,7) studies describing the relationship between health care provider recommendations and physical activity among the arthritis population are omitted.

On the basis of other evidence, we agree with the overall message (health care providers should recommend physical activity to all patients with arthritis, regardless of age), but we feel that the issues described above must be addressed before conclusions from this manuscript can be accepted as evidence for the message.

**Kristina A. Theis, MPH**
**Charles G. Helmick, MD**
**Jennifer M. Hootman, PhD**
**Kamil E. Barbour, PhD**

Arthritis Program
Centers for Disease Control and Prevention
4770 Buford Hwy
Chamblee, GA 30341
